# Function and Regulation of Protein Kinase D in Oxidative Stress: A Tale of Isoforms

**DOI:** 10.1155/2018/2138502

**Published:** 2018-04-26

**Authors:** Mathias Cobbaut, Johan Van Lint

**Affiliations:** ^1^Department of Cellular and Molecular Medicine, Faculty of Medicine, KU Leuven, Leuven, Belgium; ^2^Leuven Cancer Institute (LKI), KU Leuven, Leuven, Belgium

## Abstract

Oxidative stress is a condition that arises when cells are faced with levels of reactive oxygen species (ROS) that destabilize the homeostatic redox balance. High levels of ROS can cause damage to macromolecules including DNA, lipids, and proteins, eventually resulting in cell death. Moderate levels of ROS however serve as signaling molecules that can drive and potentiate several cellular phenotypes. Increased levels of ROS are associated with a number of diseases including neurological disorders and cancer. In cancer, increased ROS levels can contribute to cancer cell survival and proliferation via the activation of several signaling pathways. One of the downstream effectors of increased ROS is the protein kinase D (PKD) family of kinases. In this review, we will discuss the regulation and function of this family of ROS-activated kinases and describe their unique isoform-specific features, in terms of both kinase regulation and signaling output.

## 1. Oxidative Stress: Causes and Consequences

Oxidative stress is a condition that develops when the cellular redox balance is disturbed by an excessive buildup of reactive oxygen species (ROS). ROS mainly occur as a byproduct of normal cellular metabolism, due to the leak of 1–3% of electrons utilized in the mitochondrial electron transport chain for the reduction of oxygen to water, resulting in the production of superoxide [1]. Besides this “collateral” production of ROS, they are also produced deliberately. ROS (mainly H_2_O_2_) are generated by oxidases in peroxisomes, for example, during *β*-oxidation of fatty acids and flavin oxidase activity [2]. Furthermore, ROS are also produced in the endoplasmic reticulum during oxidation of maturating proteins in the ER, which helps to stabilize them during folding [3]. Another source of ROS is the production of H_2_O_2_ by nicotinamide adenine dinucleotide (NADPH) oxidase complexes (NOX) in granulocytes and macrophages to kill pathogens [4]. NOX enzymes are also activated by growth factor signaling. Via the activation of kinases and by oxidizing the active-site cysteines of Tyr and lipid phosphatases, the NOX-generated ROS can potentiate the growth factor signaling output [5–8]. While ROS produced in these contexts serve a purpose, their levels should be tightly controlled, since excessive levels of ROS can cause damage to macromolecules (such as DNA, proteins, and lipids) and cause severe mitochondrial damage, causing it to leak cytochrome c resulting in apoptosis [9–16]. To this end, cells have several antioxidant mechanisms in place to prevent the excessive buildup of ROS. These are both enzymatic (e.g., superoxide dismutases, thioredoxin reductases, and glutaredoxins) and nonenzymatic (e.g., ascorbic acid, *α*-tocopherol, and glutathione) in nature [17–20]. A disturbed redox balance is associated with a variety of pathologies, including cardiovascular disease, fibrosis, neurological disease, and cancer [21–25]. Interestingly, several cancer cell lines have been shown to harbor increased levels of ROS in comparison to nontransformed cells [26, 27]. This elevated ROS is thought to come from diverse sources: altered metabolism and mitochondrial functions, mutations in mtDNA, enhanced growth factor signaling, and activation of oncogenes such as mutant forms of Ras and c-Myc [3, 28–30]. For example, it was shown that exogenous expression of H-RasG12V in 3T3 cells increases their proliferation rate, an event dependent on increased ROS levels [31]. Furthermore it was shown that H-RasG12V increases ROS levels by activating NOX4 [32]. Besides Ras, c-Myc has also been shown to increase ROS levels in cancer cells, leading to DNA damage and genomic instability, thereby promoting cancer development [33, 34]. Moderate levels of ROS result in the activation of several signaling cascades contributing to either increased proliferation or enhanced survival of cancer cells. For example, upregulation of the PI3K/Akt pathway (e.g., via the inactivation of PTEN), MAP kinase pathways such as Erk1/2 and JNK (although the latter is also involved in ROS-induced apoptosis), and the NF-*κ*B pathway has been observed [31, 35–37]. An important downstream regulator of the oxidative stress response is protein kinase D (PKD). The mechanisms leading to the activation of different PKD isoforms in cells undergoing oxidative stress, as well as the signaling consequences of this activation will be discussed in this review.

## 2. Protein Kinase D

### 2.1. The PKD Family: Isoforms and Domain Organization

The human PKD family consists of three isoforms in humans (PKD1, PKD2, and PKD3) and belongs to the Ca^2+^/calmodulin-dependent protein kinase (CAMK) group of Ser/Thr kinases. PKD1 is the largest member, with 912 amino acids and a molecular mass of 115 kDa. PKD2 and PKD3 are smaller with molecular masses of 105 kDa and 110 kDa, respectively [38].

**Figure 1:**
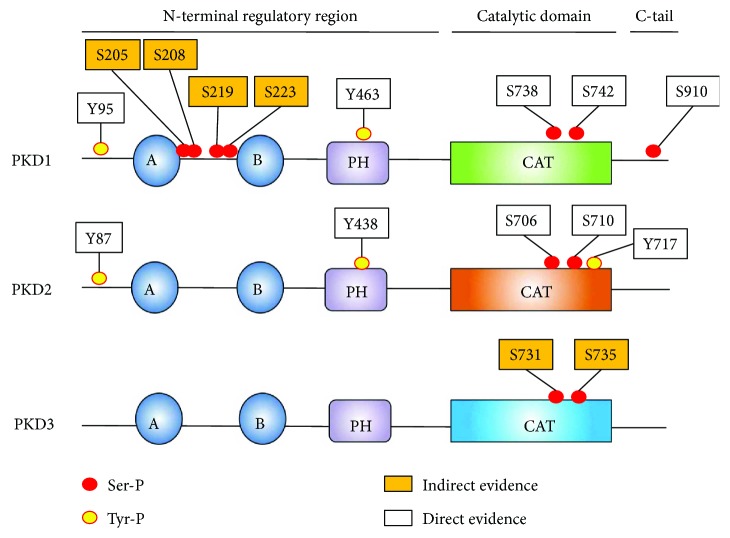
Domain organization and phosphorylation sites of PKD1/2/3 in oxidative stress conditions. Tyr phosphorylation sites are indicated with yellow-coloured circles, and Ser phosphorylation sites are indicated with red-coloured circles. “Direct evidence” indicates that the sites have been shown to be phosphorylated via immunoblotting with a site-specific antibody or via mass spectrometry. “Indirect evidence” indicates data obtained via site-directed mutagenesis or readouts dependent on general phosphorylation (such as PhosTag gels). A and B denote C1a and C1b domains, PH denotes the pleckstrin homology domain, and CAT denotes the kinase domain.

PKDs are modular enzymes that contain a long N-terminal regulatory region followed by a catalytic domain and a C-terminal extension (C-tail) (Figure 1). The N-terminal part of the protein contains several regions and domains involved in kinase autoregulation, localization, and binding to interactors.

At the extreme N-terminus, PKD1/2 contain a hydrophobic Ala(/Pro)-rich stretch (not found in PKD3), which has been hypothesized to insert in membranes [39]. This region is followed by two diacylglycerol (DAG) binding C1 domains. Because of this feature, PKDs were first classified as members of the protein kinase C (PKC) family [40]. However, the catalytic domain of PKD1 shows higher homology CAM kinases and has similar substrate and inhibitor specificity which resulted in the classification of PKDs as members of the CAMK group [41, 42]. While the C1b domain binds PDB with high affinity, DAG is preferably bound by the C1a domain [43]. In PKD, the C1a and C1b domains are separated by a large linker of approximately 70 amino acids (compared to the linker in PKC isoforms that is much shorter, for example, 8 amino acids in cPKC isoforms). Additionally, the C1a-C1b linker in PKD has important functional properties. Phosphorylation of Ser-205/208 and Ser-219/223 in the linker has been shown to generate a 14-3-3 binding site, which is crucial for ASK1 binding and downstream JNK activation in H_2_O_2_-stimulated cells (see further [44]).

The C1 domains are connected to the pleckstrin homology (PH) domain by a large linker enriched in acidic amino acids (sometimes denoted as the acidic domain). The potential regulatory role of this acidic stretch is not fully explored, but it has been suggested in an early study that it could play a role in PKD activation. This idea resulted from the observation that basic peptides and proteins (such as protamine sulfate, myelin basic protein, and histone H1) could inhibit PKD *in vitro*. On the other hand, polyanionic molecules such as heparin or dextran sulfate are capable of activating PKD, without phosphatidylserine/12-O-tetradecanoylphorbol-13-acetate (PS/TPA) [45]. Therefore, the authors hypothesized that polyanionic molecules could disrupt an intramolecular interaction between the acidic domain and a basic stretch elsewhere in the protein [45]. This hypothesis however has not been further explored. The PH domain itself functions as a negative regulator of kinase activity. A full deletion, as well as partial deletions of the PH domain, renders the kinase constitutively active [46]. Pleckstrin homology domains are known interacting modules for phosphoinositides. However, only a small number of these have lipid-binding capabilities, for which the requirements are well defined [47–49]. Structural analysis of the available NMR structures of PKD PH domains in the protein databank (2COA and 2D9Z) reveals that the PH domains of PKD lack the necessary amino acids to interact with phosphatidylinositol phosphate head groups. Hence, the PH domain of PKD does not seem to serve as a lipid-interaction module, but rather serves as a protein interaction module. Important binding partners in the context of PKD activation are G*βγ* isoforms. Binding of PKD to G*βγ* heterodimers has been proven to directly activate immunoprecipitated PKD1 *in vitro* [50]. Also, incubation of permeabilized HeLa cells with G*βγ* causes PKD activation, and when competing free PH domain was added, activation was decreased [50]. Seemingly in contrast to this finding, transfection studies showed that cotransfection of G*βγ* isoforms with phospholipase C (PLC) *β*2/3 isoforms was necessary to activate PKD. However, only certain G*βγ* isoforms could activate PKD1 and PLC*β*2/3, while other G*βγ* isoforms could activate PLC*β*2/3 but not PKD1 [51]. This indicates that structural compatibility between G*βγ* and the PH domain is required for activation of PKD, besides DAG generation by PLC*β*2/3.

The N-terminal regulatory region is followed by the catalytic domain. Notably, PKDs are non-RD kinases, that is, they do not contain an Arg in their catalytic loop (HRD motif). However, while these non-RD kinases normally are not dependent on activation loop phosphorylation, PKDs are (in most cases) dependent on activation loop Ser-738/742 (hPKD1 numbering) phosphorylation for their activity [52]. The catalytic domain is followed by a C-terminal extension (C-tail). The C-terminal portion of the tail is not conserved and may contribute to isoform-specific functions such as differential localization [53]. At the extreme C-terminus, PKD1/2 contains a PDZ-domain binding motif (type I: X-(Ser/Thr)-X-*ϕ*, where X is any amino acid and *ϕ* is a hydrophobic amino acid), which contains an autophosphorylation site [54]. The tail is likely also important in the regulation of PKD activity, since it has been shown that PKD1 C-terminal epitope tags increase *in vitro* autocatalytic activity and activity towards the peptide substrate syntide-2 compared to N-terminally tagged PKD1 [55].

### 2.2. Activation Models of PKD

#### 2.2.1. Classical PKD Activation

**Figure 2:**
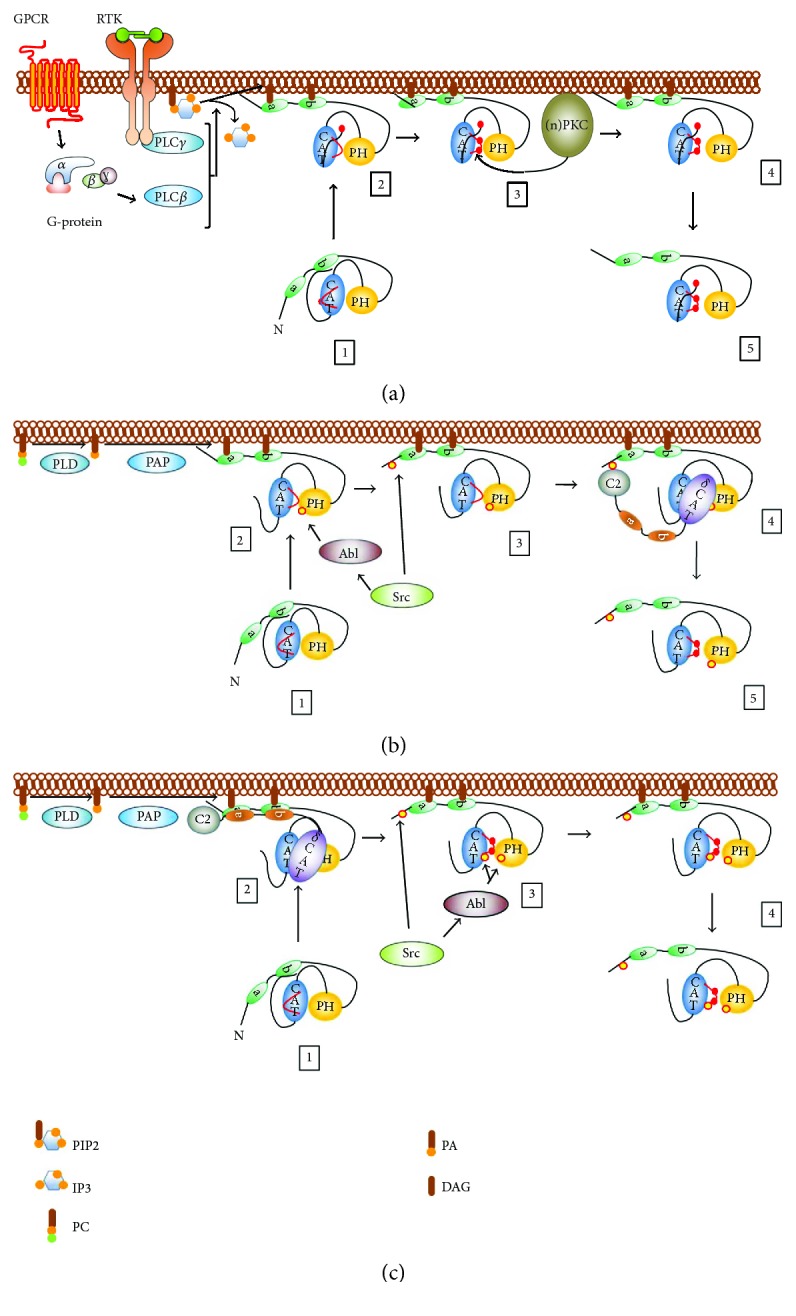
(a) Classical activation of PKD downstream of phospholipase C activity. (1) PKD1 is in an inactive resting conformation: the C1 and PH domains autoinhibit PKD activity. (2) PKD1 is recruited to DAG-containing microenvironments at the plasma membrane, which alleviates the autoinhibition exerted by the C1 domains. In this conformation, PKD has increased activity towards peptide substrates, but not towards proteins, indicative of an “unstable” open or “half-open” conformation. At this point, PKD can also exert autocatalytic activity towards Ser-910. (3) The abovementioned conformational changes and Ser-910 phosphorylation structure the kinase core for subsequent Ser-738 and Ser-742 phosphorylation by upstream PKCs. (4) Activation loop Ser phosphorylation stabilizes the PH-CAT module in an “open” conformation allowing for full PKD activity. (5) PKD1 is released from the membrane and translocates to several compartments to exert its functions. (b) Activation of PKD1 in oxidative stress conditions. (1) PKD1 is in a resting state, confer (a). (2) Activation is initiated by phosphorylation of Tyr-463 in the PH domain, which allows the recruitment of PKD to DAG generated by phospholipase D (PLD) activity at the outer mitochondrial membrane. (3) A subsequent conformational change allows for N-terminal phosphorylation at Tyr-95. (4) PKC*δ* docks to PKD1 via pTyr-95 and phosphorylates PKD1 at the activation loop Ser-738/742 residues. (5) PKD1 reaches full activity and initiates downstream signaling. (c) Activation of PKD2 in oxidative stress conditions. (1) PKD2 is in a resting state, confer (a). (2) PKD2 is recruited to DAG generated by phospholipase D (PLD) and phosphatidic acid phosphatase (PAP) activity at the outer mitochondrial membrane via its C1 domains, where it colocalizes and interacts with PKC*δ* without the need for Tyr-95 phosphorylation. PKC*δ* phosphorylates PKD2 at the activation loop Ser-706/710 residues. (3) PKD2 is phosphorylated at Tyr residues, including Tyr-87, Tyr-438, and Tyr-717, with no determined hierarchy. (4) An active and Tyr-phosphorylated PKD2 species is released from the membrane to exert its functions. PIP2: phosphatidylinositol 4,5-bisphosphate; IP3: inositol 1,4,5-trisphosphate; PC: phosphatidylcholine; PA: phosphatidic acid; DAG: diacylglycerol. Tyr phosphorylation sites are indicated with yellow-coloured circles, and Ser phosphorylation sites are indicated with red-coloured circles.

In most instances, activation of PKD begins with diacylglycerol formation at membranes (e.g., after phospholipase C activation downstream of receptor tyrosine kinase or G-protein-coupled receptor activation, Figure 2(a)), although several exceptions have been discovered [56–68]. PKD binds to local pools of DAG via its C1 domains, which results in a conformational change, abrogating an autoinhibitory mechanism. At this stage, PKD expectedly autophosphorylates at the C-tail Ser-910 residue. This idea is supported by the fact that deletion of C1a and/or C1b in PKD1 results in an increased basal autocatalytic activity towards the Ser-910 autophosphorylation site and increased activity towards peptide substrate [69]. It is noteworthy that a deletion of the C1 domains does not increase basal activity towards protein substrates, nor in an increase of Ser-738/742 autophosphorylation *in vitro* [70]. Furthermore, Ser-910 phosphorylation does not require prior activation loop Ser-738/742 phosphorylation, since a S738/742A mutant still autophosphorylates Ser-910 while substrate phosphorylation is abolished [71]. This partially activated conformation likely allows PKCs (which colocalize at DAG-containing microenvironments via their respective C1 domains) to phosphorylate PKD at the activation loop Ser-738/742 residues. This phosphorylation will in turn stabilize a conformation in which the autoinhibition by the PH domain is relieved. This has been shown in a study by Waldron and Rozengurt where PKD1 bearing nonphosphorylatable Ser to Ala substitutions in the activation loop could not be activated, but when combined with a PH domain deletion (PKD1 S738/742A ΔPH), the kinase showed high basal activity towards Syntide-2. This activity could not be further stimulated with PDB in cellulo, an enzymatic profile that is comparable to PKD1 ΔPH [72]. This indicates that the role of activation loop phosphorylation is to stabilize the active conformation after the release of the PH domain. Indeed, in an isolated catalytic domain construct, Ser-738/742 substitution with Ala has a similar activity to that with a WT PKD1 catalytic domain construct [72]. This fully active PKD species will then act locally on substrates or relocate intracellularly to exert its function. All three isoforms can be activated by DAG in an activation loop phosphorylation-dependent manner. It should be noted however that there are differences in their regulation. For example, PKD3 does not contain a C-terminal Ser autophosphorylation site. Since it has been suggested that the phosphorylation of this site primes for subsequent autophosphorylation of the second Ser site in the activation loop (i.e., Ser-742) in PKD1, it is possible that PKD3 does not autophosphorylate at this residue [71]. Furthermore, the C1 domains of the different isoforms display different affinities for DAG [43], and a deletion of the C1 domains in PKD2 results in an inactivation rather than the activating effect seen for PKD1 [73], likely pointing to differences in their activation mechanisms.

#### 2.2.2. PKD Activation in Oxidative Stress: An Isoform-Specific Matter

In oxidative stress conditions, the activation mechanism for PKD1 has historically been most studied and is well established. In contrast to classical activation by DAG downstream of G-protein-coupled receptors (GPCRs) or receptor tyrosine kinases (RTKs) through plasma membrane PLCs, activation of PKD1 in oxidative stress conditions is initiated by mitochondrial DAG production through phospholipase D (and phosphatidic acid phosphatase (PAP)) activity and generally requires the hierarchical phosphorylation of two Tyr residues in order to activate the kinase (Figures 1 and 2(b)) [74].

First, PKD1 is phosphorylated in the PH domain at Tyr-463 by Abl [75]. This initiates a conformational change, allowing for Src-mediated N-terminal phosphorylation at Tyr-95 [75, 76]. This residue is embedded in a motif that resembles the pTyr recognition motif for the C2 domain of PKC*δ*. PKC*δ* docks to the pTyr motif and consequently phosphorylates the activation loop Ser-738/742 residues, an event shown to be crucial for PKD1 activity in oxidative stress (Figures 1 and 2(b)) [76–78]. In transformed cells, Ser-910 is found not to be phosphorylated in oxidative stress conditions and thus not part of the activation mechanism [77]. Recent data indicates some remarkable isoform-specific differences in the activation mechanism during oxidative stress. Indeed, in PKD2, Tyr phosphorylation does not prime for activation loop Ser-706/710 phosphorylation, but rather the opposite: activation loop Ser phosphorylation is necessary for subsequent Tyr phosphorylation of PKD2 (Figure 2(c)) [79]. How can this divergence be explained? A possible explanation is that in PKD1, phosphorylation of Tyr-463 in the PH domain is needed to promote the deinhibited state of the PH domain-catalytic domain interaction. This allows for subsequent activation loop Ser-738/742 phosphorylation, which further stabilizes the active conformation. N-terminal Tyr-95 phosphorylation is necessary to increase the affinity of PKD1 for PKC*δ* in this step. In PKD2, Tyr kinases likely have no access to the autoinhibited conformation. Activation of the kinase is in this case fully dependent on activation loop Ser-706/710 phosphorylation to stabilize the open conformation of the kinase after DAG binding. This active form of PKD2 can then be accessed by Tyr kinases. Rather than being involved in the activation of PKD2, the role of Tyr phosphorylation in the PKD2 PH domain could be to dock specific interactors in oxidative stress, since the PH domain in PKDs acts as a protein-protein interaction hub, and the sequence surrounding the Tyr residue is in agreement with the PTB domain consensus sequence NXXY [80]. N-terminal phosphorylation in PKD2 on the other hand could be beneficial to stabilize the PKD2-PKC*δ* interaction. It should be noted however that there is no difference in the association with PKC*δ* between WT PKD2 and an N-terminal Tyr-Phe substituted mutant (PKD2 Y87F) during acute oxidative stress experiments [79]. This indicates that concentration of both kinases on DAG microenvironments on the outer mitochondrial membrane (OMM) can drive the interaction between PKC*δ* and PKD2 during acute stress, overruling the need for additional affinity contacts. However, Tyr-87 phosphorylation might be beneficial at regions of lower protein densities to increase the PKC*δ*-PKD2 affinity and to maintain PKD2 phosphorylation at the activation loop, for example, after dissociation from the OMM. In line with this, a recent study showed decreased activation loop phosphorylation of a PKD2 Y87F mutant when activated in focal adhesions [81]. Besides this differential activation mechanism, PKD2 is also differentially phosphorylated during oxidative stress. Indeed, PKD2 but not PKD1, is phosphorylated at Tyr in the P + 1 loop just before the APE motif. This is remarkable considering the fact that the activation segment is 100% conserved between the isoforms and highly conserved in all Ser/Thr kinases. This differential phosphorylation is due to a motif just C-terminal of the activation segment, which likely influences recognition by the upstream kinase c-Abl. This indicates a highly regulated signaling output towards the different isoforms in oxidative stress conditions. While no effects were seen on substrate selectivity on a peptide array, the phosphorylation of this site is shown to increase peptide substrate turnover *in vitro* [79].

PKD3 was long thought not to be activated in oxidative stress because it lacks an N-terminal Tyr residue. However, PKD3 was recently shown to be activated via oxidative stress in fibroblasts, which is reversible by treatment with the PKC inhibitor GF109203X [82]. This indicates that PKC can phosphorylate both PKD2 and PKD3 without Tyr phosphorylation at the N-terminus during acute stress.

Besides isoform-specific behavior in oxidative stress conditions, certain cell-type-specific behavior was also observed. PKD1 activation in primary neuronal cells is associated with an increase in Ser-910 autophosphorylation (Figure 1) (this is in contrast with transformed cell lines) [83]. This effect has also been shown in another study in neuronal cells, where Ser-910 phosphorylation was shown to prime for activation loop phosphorylation of PKD1 [84]. Intriguingly, PKD1 activation does not involve Tyr phosphorylation in these studies [83, 84].

### 2.3. PKD Signaling in Oxidative Stress

**Figure 3:**
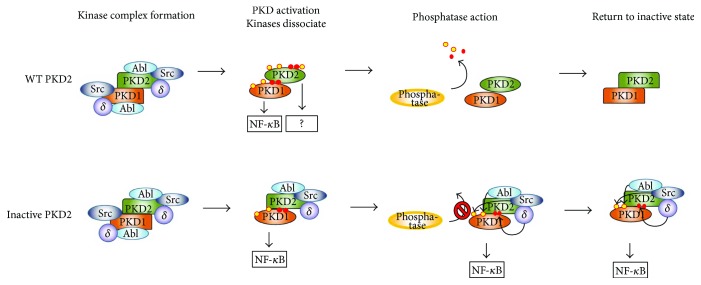
Putative function of PKD2 in NF-*κ*B signaling, based on observations with inactive PKD2. Upon exposure to oxidative stress, a kinase complex comprising dimeric PKD1/2, Src, Abl, and PKC*δ* is assembled on the outer mitochondrial membrane. In a WT PKD1/2 dimer, the upstream kinases phosphorylate PKD1/2, which causes loss of affinity and consequentially the release of upstream kinases. PKD1 and PKD2 are both activated, but only PKD1 signals to NF-*κ*B. When PKD2 cannot be phosphorylated because of mutations, the upstream kinases retain their affinity for PKD2 and can keep PKD1 phosphorylated (since they will have a similar affinity for PKD1 when it is dephosphorylated). In conjunction with this, the inactive conformation of PKD2, resulting from the fact that it cannot be phosphorylated, could protect PKD1 from phosphatase action. Both these phenomena could explain the enhanced NF-*κ*B signaling output seen with inactive PKD2 mutants. Tyr phosphorylation sites are indicated with yellow-coloured circles, and Ser phosphorylation sites are indicated with red-coloured circles.

Once activated, PKDs regulate several pathways downstream of oxidative stress. The best studied signaling output is towards the nuclear factor kappa-light-chain-enhancer of the activated B cell (NF-*κ*B) pathway. PKD1 signals to NF-*κ*B via the IKK complex, which results in the consequential degradation of I*κ*B, but the exact mechanism has not been elucidated yet [85]. NF-*κ*B activity results in upregulation of mnSOD, which detoxifies the cell from mROS, but also generates H_2_O_2_, a tumor-promoting signaling molecule [86]. Additionally, it was recently shown that ROS-induced PKD1-mediated NF-*κ*B activity results in the upregulation of epidermal growth factor receptor (EGFR) signaling components (EGFR and its ligand TGF*α*) in pancreatic cancer downstream of oncogenic Ras [87]. While PKD-induced NF-*κ*B activity in transformed cells contributes to tumor development, this pathway has recently also been shown to contribute to the physiological steady-state survival of neuronal cells. Indeed, in these cells, the PKD-NF-*κ*B-SOD2 axis is constitutively active and protects against oxidative damage [88]. In a model of excitotoxic neurodegeneration, which results in endoplasmic reticulum stress and mitochondrial dysfunction, high levels of ROS, and oxidative stress damage, the authors showed that PKD1 is rapidly deactivated after a short burst of activity, resulting in the loss of NF-*κ*B signaling and impeding neuronal survival [88]. These findings were further substantiated *in vivo* using patient samples and an ischemic mouse model [88]. Another recent study in hippocampal primary neurons showed that PKD1 is transiently activated during oxidative stress, but no changes in NF-*κ*B signaling were observed by the authors [83]. Notably, while activity and activation loop Ser-738/742 phosphorylation are necessary for signaling output to NF-*κ*B by PKD1, for PKD2, the inverse has been shown [77, 89]. Indeed, a kinase-dead PKD2 D695A (DFG to AFG) mutant strongly stimulates NF-*κ*B signaling, as do an inactivated S706/710A or Y717F mutants, while WT PKD2 (i.e., activated) or an activation loop phosphomimetic S706/710E mutant displays no increased signaling output in oxidative stress conditions [79, 89]. The reason for this isoform-specific behavior in oxidative stress is not known. One possibility is that PKD2 itself does not signal to NF-*κ*B but acts as a scaffold for PKD1, which phosphorylates an isoform-specific substrate in the NF-*κ*B pathway. Indeed, PKD1 and PKD2 are known to form heterodimers [90]. The fact that PKD2 cannot be phosphorylated by its upstream kinases through S706/710A or Y717F mutations could “trap” the upstream kinase complex (i.e., PKC*δ*, Abl, and potentially Src) on inactive PKD2. Due to dimer formation of PKD2 with PKD1, the latter is kept phosphorylated by the upstream kinases held in proximity by PKD2, potentiating its activity on NF-*κ*B (Figure 3). In addition, the inactive conformation of the PKD2 monomer in the heterodimer could protect PKD1 from phosphatases (Figure 3). It should be noted however that it is unknown whether an Asp-Ala substitution in the DFG motif of the PKD2 activation segment has an effect on the ability of PKC*δ* and Abl to phosphorylate Ser-706/710 and Tyr-717 residues. Additionally, PKD2 can potentially enhance PKD1-mediated signaling by recruiting NF-*κ*B signaling components via its PH domain in a Tyr-438 phosphorylation-dependent manner. Indeed, a phosphomimetic mutant of this residue displays increased signaling output to NF-*κ*B in the absence of H_2_O_2_ when combined with an inactivating D695A mutation, but not when combined with a S706/710E mutant [89].

Besides the effects of PKDs on NF-*κ*B, other PKD-dependent signaling functions have been described. For example, PKD1 inhibits mitochondrial depolarization and decreases cytochrome c release upon oxidative stress in mouse embryonic fibroblasts, effectively protecting them from apoptosis [91]. Interestingly, this behavior is isoform-specific, since cells expressing PKD2/3 do not display this phenotype. In hepatocytes, PKD1 has also been shown to protect cells from apoptosis by downregulating JNK signaling [92]. In epithelioid RIE-1 cells, PKD1 not only signals to NF-*κ*B but also reduces p38 phosphorylation, both of which contribute to protection from apoptosis [93]. No alterations were seen in JNK signaling in this context [93]. PKD1 also phosphorylates Hsp27 in response to oxidative stress [94, 95]. In neuronal cells, Hsp27 phosphorylation protects from ischemia-induced apoptosis by suppressing JNK activity [95].

Notably, all of the PKD1 functions described above result in prosurvival signals in oxidative stress. However, in a PKC-independent pathway, oxidative stress-induced PKD1 activity can also activate JNK downstream of death-associated protein kinase (DAPK), which results in a prodeath signal and increased necrotic cell death [96]. Furthermore, in bovine aortic ECs (BAECs), PKD1 has been shown to activate JNK via association with its upstream kinase ASK1. This association is dependent on 14-3-3 binding to PKD1, potentially via Ser-205/208 and Ser-219/223 autophosphorylation (Figure 1). The consequence of JNK activation in these cells has not been explored [44]. PKD1 also has been shown to phosphorylate Vps34 in oxidative stress [97]. Vps34 is a PI3 kinase and its phosphorylation by PKD1 leads to an activation and consequential increase of PI(3)P, resulting in increased autophagy and presumably cell death [97].

## 3. Concluding Remarks and Perspectives

The activation, regulation, and signaling properties of protein kinase D isoforms in oxidative stress come with intriguing questions that require further exploration. The remarkable isoform-specific differences point to a highly specific regulation of these kinases. Isoform-specific behavior in oxidative stress is also seen for other kinase families. For example, Akt isoforms are differentially regulated in oxidative stress, where Akt2 is specifically inactivated after H_2_O_2_ stimulation via the generation of a disulfide bond [98]. Besides their differential regulation, PKD isoforms also display distinct signaling properties. A striking example of this is their different signaling output to NF-*κ*B. While activated PKD1 in oxidative stress signals to NF-*κ*B, it has been shown that for PKD2, inactive forms stimulate the NF-*κ*B signaling output [79, 89]. However, the functional relevance of inactive PKD2 is unclear, since it is also activated during oxidative stress, likely to an even larger extent than PKD1 [79]. The role of WT PKD2 in NF-*κ*B signaling as part of a PKD1-PKD2 heterodimer is likely twofold: (1) to recruit NF-*κ*B signaling components via its PH domain and (2) to enhance complex formation with upstream kinases to enhance PKD1 activation. An important question is whether there are other, currently unknown pathways activated PKD2 contributes to in oxidative stress.

Isoform-specific signaling behavior in redox signaling is also observed within the PKC family. For example, in the protection of the heart from ischemic events, the related nPKCs PKC*δ* and PKC*ε* play opposing roles, with PKC*ε* being cardioprotective while PKC*δ* increases damage induced by ischemia both *in vitro* and *in vivo* [99].

Another level of complexity lies within cell-type-specific behavior of PKDs in oxidative stress, both in their regulation and in their signaling properties. For example, as mentioned before, in neuronal cells, PKD1 activation does not always involve Tyr phosphorylation, but rather Ser-910 phosphorylation, and it does not contribute to an increased NF-*κ*B signaling output in these cells [83, 84]. Moreover, a recent study shows a loss of homeostatic NF-*κ*B signaling output during increased oxidative stress due to a rapid downregulation of PKD1 activity [88].

In conclusion, future studies should be carefully carried out to dissect the ROS-mediated regulation and functional roles of the individual PKD isoforms in different cell types, in order to understand the full extent of PKD-mediated signaling in oxidative stress.
